# Comparison of Urethrocutaneous Fistula Rate After Single Dartos and Double Dartos Tubularized Incised Plate Urethroplasty in Pediatric Hypospadias

**DOI:** 10.7759/cureus.13378

**Published:** 2021-02-16

**Authors:** Fatima Naumeri, Malik Asad Munir, Hafiz Mahmood Ahmad, Muhammad Sharif, Nukhbat U Awan, Ghazala Butt

**Affiliations:** 1 Pediatric Surgery, King Edward Medical University/Mayo Hospital, Lahore, PAK; 2 Otolaryngology, King Edward Medical University/Mayo Hospital, Lahore, PAK; 3 Dermatology, King Edward Medical University/Mayo Hospital, Lahore, PAK

**Keywords:** dartos flap, hypospadias, tubularized incised plate urethroplasty, snodgrass urethroplasty, urethrocutaneous fistula, single dartos flap, double dartos flap

## Abstract

Background and objective

Tubularized incised plate (TIP) urethroplasty is an easy and popular technique for repairing hypospadias, however urethrocutaneous fistula (UCF) is a frequently reported complication. Different techniques are used to reduce this complication. We aimed to compare the rate of UCF after single dartos and double dartos TIP urethroplasty in children with distal and mid penile hypospadias.

Methods

A randomized controlled trial (NCT 04699318) was conducted in the Department of Pediatric Surgery, Mayo Hospital, Pakistan from August 2017 to February 2018, after ethical approval. After informed consent, a total of 60 patients with distal and mid penile hypospadias who were uncircumcised, had no chordee, and/or previous surgery, were randomly allocated in two groups using computer generated table numbers. Group A underwent single dartos TIP urethroplasty and Group B underwent double dartos TIP urethroplasty. Catheter was removed on day 10 post-operatively in both groups and primary outcome (UCF) was noted after a week of catheter removal. Rate of UCF was compared using chi square and p-value of <0.05 was taken as significant. Data was stratified to check for effect modifiers.

Results

Out of 60 children, eight (13.3%) developed UCF. In Group A, seven (23.3%) developed UCF and in Group B, one (3.3%) developed UCF (p-value 0.02). In both groups, no patient (0%) had urethral disruption, penile torsion, skin necrosis or meatal stenosis.

Conclusion

Additional covering of neo-urethra by a double dartos layer significantly reduces fistula rate after tubularized incised plate urethroplasty in both primary distal and mid penile hypospadias.

## Introduction

Hypospadias is a congenital condition in which urethra opens on the penile ventral aspect, anywhere from glans to perineum and may be associated with chordee [1,2]. Although incidence of hypospadias is one in every 250 male births, reported prevalence ranges from 2.1 to 39.1 per 10,000 births globally, and is higher in cases of positive family history [1,3]. The incidence also varies regionally, and is increasing globally at the rate of 0.25 cases per 10,000 births/year [3,4].

Hypospadias is repaired by several techniques [1,5]. Techniques include single stage procedures like meatal advancement with glanuplasty incorporated (MAGPI), the glans approximation procedure (GAP), primary tubularization, the Mathieu or flip-flap, the Duplay or primary tubularization with the incision of the urethral plate (TIP) or staged procedures like Bracka urethroplasty [1].

Post urethroplasty major complications include urethrocutaneous fistula, hematoma, skin necrosis, meatal stenosis and urethral disruption, but the most common complication is urethrocutaneous fistula with overall range from 0% to 35% [1,6].

The causes of post urethroplasty fistula include local factors like infection, local ischemia, an improper technique, poor tissue handling, distal obstruction due to meatal stenosis, severity of hypospadias, patient factors, everted edges of epithelium in neourethra, and prolonged tourniquet. Systemic factors causing immunodeficiency and nutritional factors are also implicated [6]. To reduce the rate of urethrocutaneous fistula, different techniques have been used and one of these is tissue inter-positioning of dartos fascial flap between the neourethra and skin [7].

Dartos fascial flap can be used as a single layer or as a double layer. In a single dartos fascial flap technique, flap of dartos fascia is raised from preputial skin on dorsal aspect and applied on neourethra as a single layer, but in double dartos flap technique, the flap is divided into two equal halves and then double breasting on top of neourethra [8].

There is need for randomized controlled trials to establish the superiority of double dartos urethroplasty. According to the systematic review for a distal penile hypospadias repair, double layer dartos flap had lower rate of urethrocutaneous fistula (0.6%) than single layer dartos flap (5.1%). This systematic review however, lacks evidence in mid-penile hypospadias [9]. Based on this gap, we compared urethrocutaneous fistula rates in single dartos and double dartos TIP urethroplasty in children with primary distal and mid penile hypospadias.

## Materials and methods

This randomized controlled trial (NCT 04699318 and IRB No 223/RC/KEMU dated 27/03/2017) was carried out in department of Pediatric Surgery, King Edward Medical University/Mayo Hospital Lahore from August 2017 to February 2018. Sample size of 60 patients (30 in each group) was estimated by using 95% confidence level with 80% power of test with expected percentage of urethrocutaneous fistula in double layer as 0.7% and single layer as 26% [10]. Non-probability consecutive sampling was used.

All the boys aged 1 to 12 years presenting in Pediatric Surgery Department, with both distal and mid penile hypospadias were included in the study. Exclusion criteria included presence of severe chordee, previously operated cases, patients with undescended testis and/or circumcision. Children fulfilling inclusion and exclusion criteria were enrolled in the study after informed verbal and written consent was obtained from parents about sharing of data, blinding about definitive procedure, details of procedures, post-operative complications, and follow-up. Children were randomly allocated in two groups via computer generated tables.

**Figure 1:**
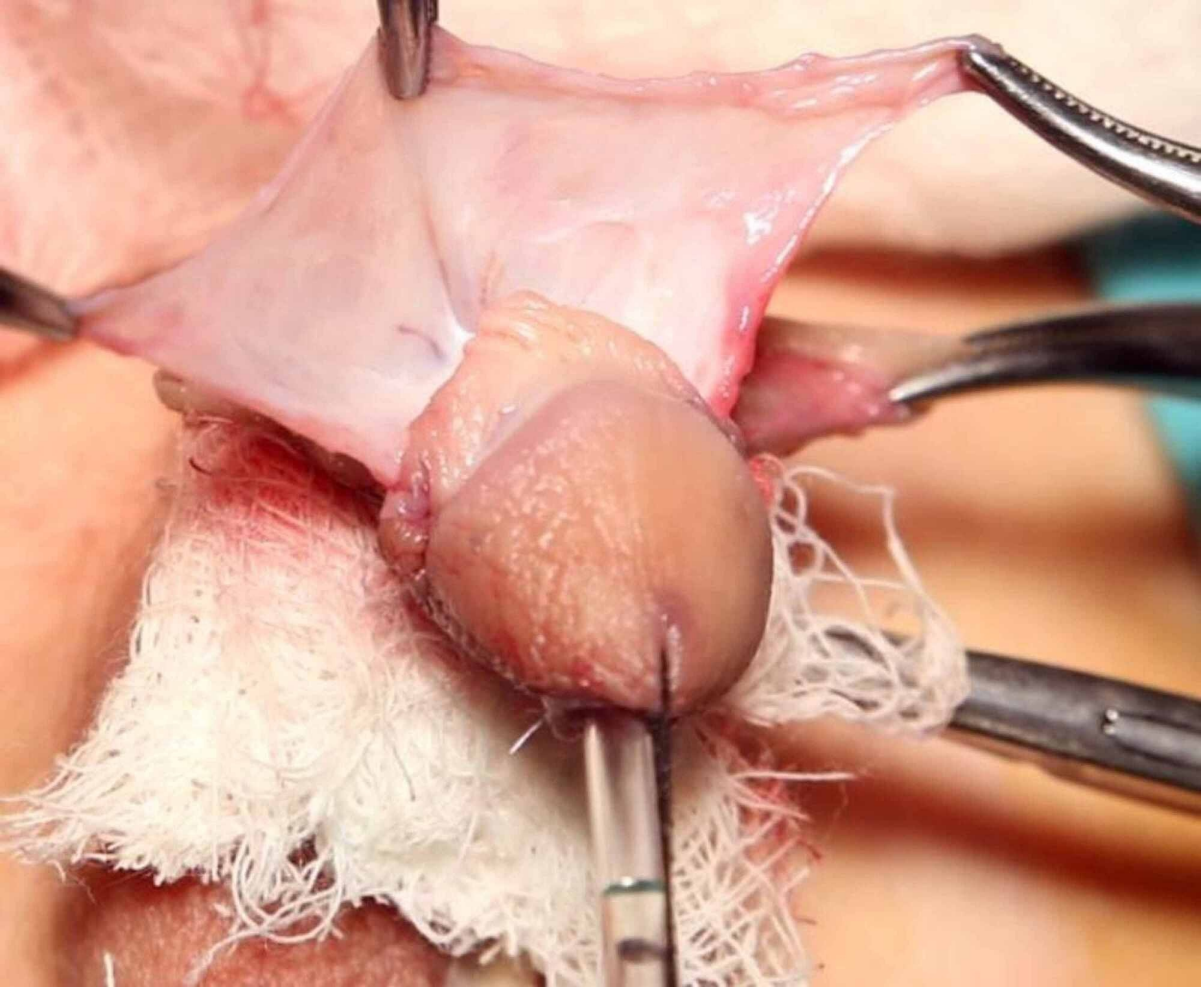
Formation of preputial dartos flap on dorsal aspect.

**Figure 2:**
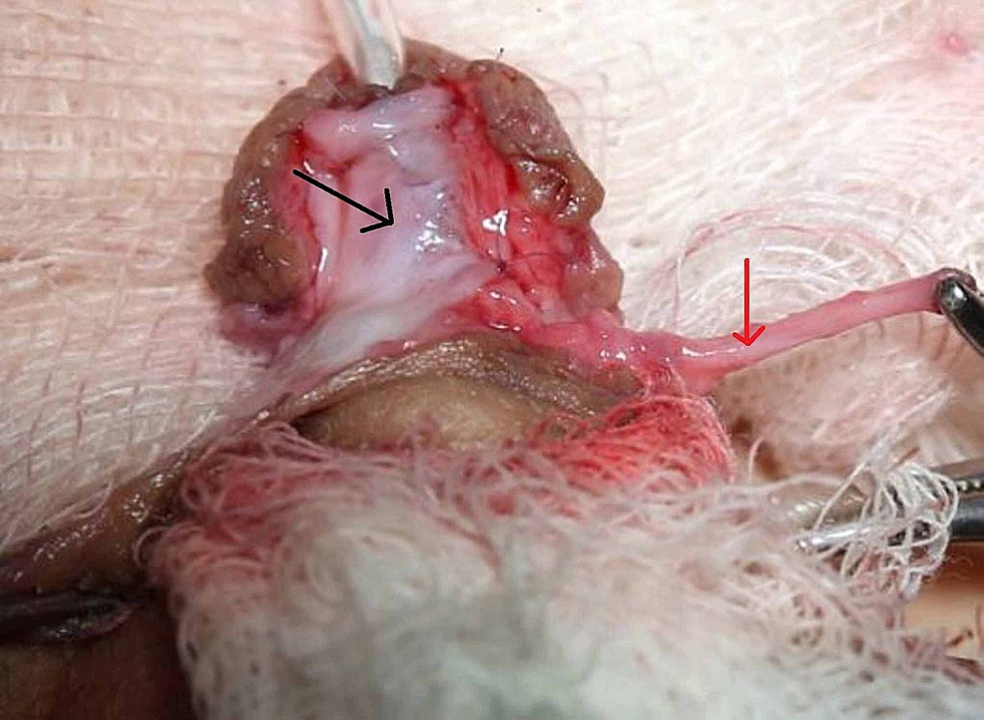
Double breasting of preputial dartos flap on neo-urethra in group ‘B’. Black Arrow: First layer of preputial dartos flap covering the neo-urethra Red Arrow: Second layer of preputial dartos flap to be double breasted on first Layer

Children in group-A underwent single dartos urethroplasty and in group-B underwent double dartos urethroplasty. Under general anesthesia, stay suture was placed on glans and penile tourniquet was applied. Penis was degloved, deep longitudinal incision was made in groove, and neo-urethra was made over an age appropriate nelaton catheter, using polydioxanone suture (PDS 6/0 or PDS 7/0). This neo-urethra was covered with dartos fascial flap, raised from preputial skin on dorsal aspect (Figure 1). In Group ‘A’, dartos flap was placed as a single layer using button-hole technique for transposition, while in Group ‘B’, dartos flap was divided in two equal halves and then double breasted on neo-urethra (Figure 2). Intravenous antibiotics were given for five days. Children were kept in ward for 10 days post operatively, after which catheter was removed. Children were called after seven days in accordance with the ward routine and were assessed for urethrocutaneous fistula (abnormal communication between urethra and penile skin, confirmed clinically by two or more streams during micturition) and other post-operative complications like total disruption (breakdown of neo-urethra resulting in urination from previous urethral meatus), penile torsion (clinically penile rotation on its axis), skin necrosis and meatal stenosis (narrow meatus causing dysuria and thin urinary stream).

The data was collected by the doctor on duty in the outdoor department on a prescribed pro-forma. Initial group allocation was not known to the doctor. Data were entered and analyzed on SPSS version 23.0 (IBM Corp., Armonk, NY, USA). Quantitative variable like age, weight was presented as mean and standard deviation. Qualitative variable like urethrocutaneous fistula, total disruption, presence of penile torsion, meatal stenosis was presented as frequency and percentage. Comparison of two groups was done by applying chi-square test. A p-value <0.05 was taken as significant. Data were stratified for age, weight and type of hypospadias to address effect modifiers. Post stratification, chi-square test was applied and p-value <0.05 was considered as statistically significant.

## Results

Total 60 patients were enrolled. Mean age of patients in group ‘A’ was 6.1 ± 4.1 years and in group ‘B’ was 7.8 ± 3.4 years. Distal hypospadias was present in 48 (80%) patients and mid penile hypospadias was present in 12 (20%) patients. Overall comparison of different age and weight stratification along with different types of hypospadias in both groups is shown in Table 1.

**Table 1:** Comparison of type of hypospadias, age, and weight stratification in both groups.

VARIABLES		GROUP A	GROUP B
AGE (years)	1-4	12 (40%)	7 (23.3%)
	5-8	6 (20%)	8 (26.7%)
	9-12	12 (40%)	15 (50%)
WEIGHT (kg)	7-12	8 (26.7%)	8 (26.7%)
	13-17	8 (26.7%)	11 (36.7%)
	>18	14 (46.7%)	11 (36.7%)
HYPOSPADIAS TYPE	Distal penile	21 (70%)	27 (90%)
	Mid penile	9 (30%)	3 (10%)

Urethrocutaneous fistula developed in seven (23.30%) patients in group ‘A’ (single dartos TIP) while in one (3.3%) patient in group ‘B’ (double dartos TIP) with p-value of 0.02. Table 2 shows effect of location of hypospadias on formation of fistula. Age and weight stratification revealed no difference in fistula formation in both groups (p-value >0.05). In both groups, no patient (0%) had urethral disruption, penile torsion, skin necrosis or meatal stenosis.

**Table 2:** Hypospadias type and urethrocutaneous fistula between groups. TIP: Tubularized incised plate

Hypospadias location	Urethrocutaneous fistula	Groups		Total	p-value
		Single dartos TIP (A)	Double dartos TIP (B)		
Distal penile	Present	5 (23.8%)	1 (3.7%)	6 (12.5%)	0.03
	Absent	16 (76.2%)	26 (96.3%)	42 (87.5%)	
Mid penile	Present	2 (22.2%)	0 (0%)	2 (16.7%)	0.37
	Absent	7 (77.8%)	3 (100%)	10 (83.3%)	

## Discussion

In this study, tubularized incised plate (Snodgrass) urethroplasty was used for distal and mid penile hypospadias. Since 1932, when Mathieu first described a peri-meatal flap for repairing distal penile hypospadias, many surgical techniques have been used [11]. Snodgrass urethroplasty basically involves a deep longitudinal incision in the entire length of groove to facilitate a tension-free tubularization and a vertical meatus. However, despite this modification to decrease complications, urethrocutaneous fistula is still reported [12].

Suture material, infection, previous operations, catheterization, surgical skills like tension-free anastomosis and prevention of epithelium eversion, all are implicated in prevention of fistula formation [6,13]. Another important factor in fistula prevention is the use of intermediate layer between neourethra and skin. Retik was first to use preputial dartos flap as an intermediate layer. In cases of reoperation or circumcised children, preputial dartos flap is not available. The other options available are external spermatic fascia, tunica vaginalis, scrotal or ventral dartos flap [1,13]. In this study preputial dartos flap was used, as we excluded circumcised and previously operated cases.

Snodgrass transposed the preputial dartos flap ventrally in a button-hole fashion over the neo-urethra. The reason was to avoid penile torsion in case of asymmetrical flap [12]. In this study, we transposed single dartos flap using the button-hole technique, while in group B, the dartos flap was divided into two equal halves and double breasted on top of neo-urethra.

Kamal reported that covering neo-urethra with double dartos flap was superior to single dartos flap for preventing fistula. The reason for higher fistula rates after single dartos flap was its micro-perforation due to dissection injury or flap infection. The other benefit reported by him was lack of torsion in cases of double dartos flap [14].

In this study, seven (23.3%) patients in group A and one (3.3%) patient in group B developed urethral fistula with a p-value of 0.02, which is statistically significant. This finding is supported by the literature. Abolyosr performed double dartos urethroplasty in 158 boys and reported no fistula [15]. Similarly Erol et al. reported that double dartos was more effective in preventing fistula formation [16]. Mustafa et al. performed double dartos in 28 boys and reported no fistula [17]. In a study by Yiğiter et al., fistula rate after double dartos urethroplasty was 0.7%, while it was 26% with single dartos urethroplasty and 29.4% after standard TIP urethroplasty [10].

In this study, using double dartos urethroplasty we were able to reduce fistula more in cases of distal penile hypospadias (p-value 0.03). Only one patient (3.7%) developed fistula in double dartos urethroplasty for distal penile hypospadias as compared to five patients (23.8%) in single dartos urethroplasty. Similarly there was no fistula in double dartos urethroplasty for mid penile hypospadias as compared to two patients (22.2%) in single dartos urethroplasty. A systematic review recently also documented lower rate of fistula formation in distal penile hypospadias repaired with double dartos 0.6% versus 5.1% after single dartos flap urethroplasty in primary hypospadias, while tunica vaginalis flap was more effective in re-operation [9]. Similarly another multicentric study [18] revealed that double dartos urethroplasty had a fistula rate of 1.01% (four children in 394 operated cases).

The other complications reported in literature include meatal stenosis, urethral stricture, penile skin necrosis, penile torsion, urethral diverticulum, and total urethral disruption [1,5,7,18]. In this trial, no patient developed the other reported complications. A recent study involving 25 patients of distal penile hypospadias and 18 patients of mid penile hypospadias evaluated causes of urethral fistula post urethroplasty and found that longer urethral length, meatal stenosis, and pyuria resulted in greater fistula formation [19]. In this study we electively operated when there was no pyuria and urethral length did not affect the outcome (six out of 48 children with distal penile hypospadias and two out of 12 children with mid penile hypospadias suffered from fistula).

Limitation is that it is slightly underpowered study (80% power) involving a single center and relatively small sample size. Future trials overcoming these limitations are needed to develop guidelines.

## Conclusions

Additional covering of neo-urethra by a double dartos layer significantly reduces fistula rate after tubularized incised plate urethroplasty in both primary distal and mid penile hypospadias. With the advent of more expensive therapies like platelet rich plasma, stem cells, and other options for intermediate layer, we recommend the routine use of cost effective double dartos preputial flap in selected children with primary hypospadias.
